# A Neurosemantic Theory of Concrete Noun Representation Based on the Underlying Brain Codes

**DOI:** 10.1371/journal.pone.0008622

**Published:** 2010-01-13

**Authors:** Marcel Adam Just, Vladimir L. Cherkassky, Sandesh Aryal, Tom M. Mitchell

**Affiliations:** 1 Department of Psychology, Carnegie Mellon University, Pittsburgh, Pennsylvania, United States of America; 2 Machine Learning Department, School of Computer Science, Carnegie Mellon University, Pittsburgh, Pennsylvania, United States of America; Indiana University, United States of America

## Abstract

This article describes the discovery of a set of biologically-driven semantic dimensions underlying the neural representation of concrete nouns, and then demonstrates how a resulting theory of noun representation can be used to identify simple thoughts through their fMRI patterns. We use factor analysis of fMRI brain imaging data to reveal the biological representation of individual concrete nouns like *apple*, in the absence of any pictorial stimuli. From this analysis emerge three main semantic factors underpinning the neural representation of nouns naming physical objects, which we label *manipulation*, *shelter*, and *eating*. Each factor is neurally represented in 3–4 different brain locations that correspond to a cortical network that co-activates in non-linguistic tasks, such as tool use pantomime for the *manipulation* factor. Several converging methods, such as the use of behavioral ratings of word meaning and text corpus characteristics, provide independent evidence of the centrality of these factors to the representations. The factors are then used with machine learning classifier techniques to show that the fMRI-measured brain representation of an individual concrete noun like *apple* can be identified with good accuracy from among 60 candidate words, using only the fMRI activity in the 16 locations associated with these factors. To further demonstrate the generativity of the proposed account, a theory-based model is developed to predict the brain activation patterns for words to which the algorithm has not been previously exposed. The methods, findings, and theory constitute a new approach of using brain activity for understanding how object concepts are represented in the mind.

## Introduction

How a simple concept like *apple* is represented in the human mind has been of interest to philosophers for centuries, but the question has not been amenable to scientific approaches until recently. The emerging technologies of brain imaging have now made it possible to examine the *neural* representation of such concepts in the human brain, in a way that has been revealing of the mental content. It is clear that the neural representation of such concepts involves multiple brain areas specialized for various types of information, indicating that the representations can be decomposed into components. In the case of discrete physical objects, the neural representations can be related to verbs of perception and action that apply to the objects [1]. For example, *apple* appears to be neurally represented in terms of an apple's visual properties, graspability, purpose, etc., and the representation is distributed across a number of relevant brain areas; for example, the information about the physical actions that can be applied to an object are represented in cortical areas related to control of hand actions [1], [2].

A central issue addressed here concerns the underlying semantic dimensions of representation of concrete nouns and the physical objects to which they refer. What are the underlying semantic, psychological, or neural dimensions in terms of which *apple* is represented? To take a simpler example, a kinship term such as *grandmother* is likely to be represented in terms of gender (female), generation level relative to a reference person (two generations older), and lineality (direct ancestor) [3]. The dimensions of kinship terms are easier to discern because of the well-structured biological and social domains to which they refer. The corresponding representational dimensions of *apple* are far less clear. However, new methods of neuroimaging and machine learning have the potential of revealing the dimensions of representation that the brain uses.

A new approach, combining fMRI neuroimaging and machine learning techniques, successfully characterized the neural representations of physical objects like apples [1]. This approach proposed that meanings of physical objects can be characterized in terms of 25 features, namely the nouns' co-occurrence frequencies with 25 verbs of perception and action in a large text corpus. For example, one semantic feature (independent variable in a regression model) was the frequency with which the noun co-occurs with the verb *taste*. The farthest reaching contribution of this model was its generativity, enabling it to extrapolate sufficiently to predict the neural representation (fMRI-measured brain activity) of words that were new to the model, simply on the basis of (1) the new words' co-occurrence frequencies with the 25 verbs, and (2) the weights associating those frequencies to patterns of brain activation in response to a fixed number of words. When presented with previously unseen brain activation patterns generated by two new concrete nouns, the model was able to correctly match the two nouns to the two patterns 77% of the time, very far above chance level.

The relative success of this previous model speaks to the choice of verbs used for co-occurrence measures. Many of the verbs pertain to physical manipulation such as *touch*, *rub*, *lift*, *manipulate*, *push*, and *move*. Some pertain to eating: *taste*, *eat*. The set of verbs was generated intuitively as actions and perceptions that seemed applicable to physical objects.

The current study takes a different approach, asking whether there is some bottom-up analytic procedure that reveals the underlying dimensions of representation, perhaps more compactly. Is there a set of fundamental neural dimensions that arise in the representation of physical objects that such a procedure can reveal?

There is a rich history of applying dimension-reduction techniques, such as factor analysis and multidimensional scaling, to behavioral data to recover the underlying dimensions of meanings of words, including classic studies of color terms [4], verbs of motion [5], animals [6], and a variety of different domains [7]. Here we apply factor analysis to neural data obtained with fMRI to determine the semantic factors underlying the brain activation. To foreshadow our results, we found that factor analysis indicated three fundamental semantic dimensions of neural representation of the physical objects in the 60-item stimulus set.

A second innovation of this study is its exclusive focus on the representation of words rather than on pictures of objects. Much of the previous research has focused on or included visual depictions of the objects of interest, rather than focusing on words (Mitchell et al. [1] presented word-picture pairs). Various kinds of depictions (such as line drawings or photographs) inherently present a particular instantiation of a given object category, and they explicitly depict some of the object's visual features, which in turn are represented in the perceiver's brain. By contrast, words are symbols whose neural representations are entirely retrieved from previous knowledge rather than being at least partly visually perceived.

Which particular brain locations are involved in the representation of a concept depends in part on how the concept is evoked. Previous neuroimaging studies that presented a visual depiction of the object, such as a line drawing or photograph, have determined which specific brain areas play a role in the high-level visual representation of categories of physical objects (such as faces), indicating that there is a set of areas, particularly in ventral temporal cortex, that respond differentially to *pictures* of a set of disparate categories of objects, such as houses, faces, and chairs [8]–[10]. Moreover, by applying machine learning or pattern-based classification methods to fMRI data (reviewed in [11]–[13]), such studies have succeeded in finding a mapping between multivariate patterns of brain activity and a given object category. The remarkable successes in identifying the brain activity associated with viewing classes of visual depictions of objects has focused, unsurprisingly, on the brain's primary and secondary (ventral temporal) visual areas. Here, with printed words as stimuli, we ask if it is possible to identify higher order cortical representations (in addition to the perceptual representations) of the semantic properties of a concrete noun. Moreover, we attempt to specify the cortical locations at which the different semantic factors are processed.

A third innovation of this study lies in its examination of the commonality of the neural representation of words across different people. Only recently has it been possible to demonstrate that there is a great deal of commonality across people in their neural representations of visually depicted objects, like *screwdriver*, *drill*, *hut*, or *castle* [14]. Here we examine the commonality of the representation of concrete nouns across people. The measure of commonality is whether a classifier (a mathematical function that here maps from fMRI activation patterns to word labels), trained on the brain activation patterns of a set of people, can accurately classify (label) patterns of activation obtained from people outside of that set. Although the issue of cross-person commonality of representation is dealt with succinctly, it yields one of the most far-reaching conclusions of this research, indicating whether one person's neural representation of the meaning of a concrete noun closely resembles another person's.

The machine learning or pattern classification approach is also used in a more fundamental way, namely for determining whether a neural signature of each word's meaning, derived from a subset of a given participant's data, can be used to classify (label) the words from an independent subset of that same participant's brain activation data. The classification approach is used to assess how well the factor analysis output characterizes individual neural representations.

The findings reported here thus constitute several types of advances. The central focus concerns the semantic organization of the neural representation of familiar concrete objects, revealing the component building blocks of the brain's representation of the meaning of physical objects. Second, we report the neural representation evoked by words rather than pictures. A third novel aspect of the findings is the discovery of significant cross-participant commonality in neural representations of word meaning, such that the activation patterns of an individual participant can be identified based on training data drawn exclusively from other people. Finally, we demonstrate the generativity of the proposed principles, allowing a model to predict the activation of a new concrete noun based on its semantic properties.

## Materials and Methods

### Participants

Eleven adults (eight right-handed females, two clearly right-handed males, and one male with right-handedness for tool use, with all 11 participants showing left-dominant activation) from the Carnegie Mellon community participated and gave written informed consent approved by the University of Pittsburgh and Carnegie Mellon Institutional Review Boards. Eight additional participants were excluded because of either excessive head motion (two participants) or insufficient stability of voxel activation profiles (six participants).

### Experimental Paradigm

The stimuli were 60 words, containing five exemplar concrete objects from twelve taxonomic categories: body parts, furniture, vehicles, animals, kitchen utensils, tools, buildings, building parts, clothing, insects, vegetables, and man-made objects, as shown in Table 1. The 60 words were presented six times (in six different random permutation orders). Each word was presented for 3s, followed by a 7s rest period, during which the participants were instructed to fixate on an X displayed in the center of the screen. There were twelve additional presentations of a fixation X, 31s each, distributed across the session to provide a baseline measure.

**Table 1:** 60 stimulus words grouped into 12 semantic categories.

Category	Exemplar 1	Exemplar 2	Exemplar 3	Exemplar 4	Exemplar 5
body parts	leg	arm	eye	foot	hand
furniture	chair	table	bed	desk	dresser
vehicles	car	airplane	train	truck	bicycle
animals	horse	dog	bear	cow	cat
kitchen utensils	glass	knife	bottle	cup	spoon
tools	chisel	hammer	screwdriver	pliers	saw
buildings	apartment	barn	house	church	igloo
building parts	window	door	chimney	closet	arch
clothing	coat	dress	shirt	skirt	pants
insects	fly	ant	bee	butterfly	beetle
vegetables	lettuce	tomato	carrot	corn	celery
man-made objects	refrigerator	key	telephone	watch	bell

### Task

When a word was presented, the participants' task was to actively think about the properties of the object to which the word referred. To promote their consideration of a consistent set of properties across the six presentations of a word, they were asked to generate a set of properties for each item prior to the scanning session (for example, the properties for the item *castle* might be *cold*, *knights*, and *stone*). Each participant was free to choose any properties for a given item, and there was no attempt to impose consistency across participants in the choice of properties.

### fMRI Procedures

Functional images were acquired on a Siemens Allegra (Erlangen, Germany) 3.0T scanner at the Brain Imaging Research Center of Carnegie Mellon University and the University of Pittsburgh using a gradient echo EPI pulse sequence with TR = 1000 ms, TE = 30 ms and a 60° flip angle. Seventeen 5-mm thick oblique-axial slices were imaged with a gap of 1 mm between slices. The acquisition matrix was 64×64 with 3.125-mm×3.125-mm×5-mm voxels. Initial data processing was performed using SPM2 (Wellcome Department of Cognitive Neurology, London).

### Data Preprocessing

The data were corrected for slice timing, motion, and linear trend, and were normalized into MNI space without changing voxel size (3.125×3.125×6 mm). The gray matter voxels were assigned to anatomical areas using Anatomical Automatic Labeling (AAL) masks [15]. For some analyses, the gray matter voxels were partitioned into five bilateral brain areas or “lobes” using AAL masks: frontal, parietal, temporal, occipital, and an idiosyncratically-defined fusiform “lobe” which included the fusiform and parahippocampal gyri. This fusiform “lobe” was separated from the other areas because of its prominence in previous studies of object representations. (The temporal and occipital “lobes” are hence also idiosyncratically-defined because their definition excludes their usual share of the fusiform and parahippocampal gyri.) A later check found no voxels relevant to the reported outcomes outside of the five lobes.

The percent signal change relative to the fixation condition was computed at each gray matter voxel for each stimulus presentation. The main input measure for the subsequent analyses consisted of the mean of the four brain images acquired within a 4s window, offset 4s from the stimulus onset (to account for the delay in hemodynamic response). The intensities of the voxels in this mean image for each word were then normalized (mean = 0, SD = 1).

### Selecting Voxels with Stable Activation Patterns

The analyses below generally focused on a small subset of all the voxels in the brain, namely those whose *activation profile* over the 60 words was *stable* across the multiple presentations of the set of words. The assumption here is that the activation levels of only the relatively stable voxels provide information about objects. A voxel's stability was computed as the average pairwise correlation between its 60-word activation profiles across the multiple presentations that served as input for a given model (the number of presentations over which stability was computed was four or six, depending on the analysis). Here the *60-word activation profile* of a voxel for a particular presentation refers to the vector of 60 responses of that voxel to the words during that presentation. A stable voxel is thus one that responds similarly to the 60 word stimulus set each time the set is presented.

### Factor Analysis Methods

To factor the neural activity associated with the 60 different word stimuli into different components shared across participants and brain lobes, we used a two-level exploratory factor analysis based on principal axis factoring with varimax rotation, using the same algorithm as the SAS factor procedure (www.sas.com).

At the first level, a separate factor analysis was run on each lobe of each participant, using as input the matrix of intercorrelations among the activation profiles of the 50 most stable voxels in the lobe. (Prior to computing the intercorrelations, the voxels' activation profiles within each lobe and participant were averaged over six presentations and normalized over the 60 words to have a mean = 0 and SD = 1. The choice of the particular number of voxels (50) used as input was motivated by similar analyses in other datasets where 50 was the smallest number of voxels that maximized classification accuracy.) The goal of each of these first-level factor analyses was to reduce the data from the activation profiles of many (50) stable voxels to a few factors that characterized the profiles of most of the stable voxels in each lobe of each participant.

Then a second-level factor analysis was run to identify factors that were common across lobes and participants, a procedure known as higher-order factor analysis [16]. (The search for commonality across lobes was motivated by the assumption that a semantic factor would be composed of a large-scale cortical network with representation in multiple brain lobes.) The input to the second-level analysis consisted of the five dominant first-level factors obtained from each lobe of each participant. (Below this two-step factor analysis is compared to a single-level analysis.)

To define the factor analysis models precisely, we introduce the following notation:

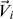
 is the 60-word mean activation profile of the i-th voxel (*i* = 1…50)

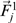
 is the first-level factor profile over 60 words of the j-th factor (*j* = 1…5)

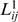
 is a first-level loading of *i*-th voxel on factor 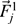

Then the following equation defines voxel activation profiles as a linear combination of factor profiles and serves as a model for the first-level factor analysis.
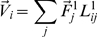



After the first-level factor analysis is computed, we have the matrix of first-level loadings 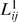 (and we also have voxel profiles 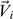 for all 50 voxels in a lobe). The equations above (corresponding to *i* = 1…50) can be solved for the unknown factor profiles 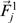 (using least squares), producing five first-level factor profiles. These factor profiles (which apply to all 50 voxels within that lobe of that participant) constitute the factor scores for each of the 60 words.

This algorithm was applied separately for each set of 50 voxels selected from five lobes of four participants, resulting in 20 first-level factor analyses. (The motivation for choosing four participants is given below.) The five dominant factors were selected from each of these first-level analyses, to produce a set of 100 first-level factors 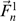, where *n* = 1…100. The choice of five factors from each first-level analysis was based on observing that the first five factors had eigenvalues greater than one, and that additional factors typically produced diminishing returns in characterizing the voxel activation profiles. These 100 first-level factors were used as input to the second-level factor analysis.

Now define 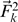 as a second-level factor profile over 60 words (*k* = 1…10), 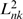 as a second-level loading of *n*-th first-level factor on second-level factor 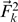.

Then the following equation defines the first-level factor profiles as a linear combination of second-level factor profiles and serves as a model for the second-level factor analysis.
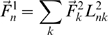



The second-level factor analysis produces a matrix of second-level 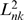 loadings, and we also have the first-level factor profiles 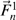. The number of factors to which the analysis was limited was 10 and of these 10 second-level factors, only the first four factors were common to all four of these participants. Solving the above equation for the unknown second-level factor profiles (using least squares) produces the vectors of second-level factor profiles over the 60 words. The factor profiles from these four factors constitute the factor scores for each word.

Factor loading matrices from all first-level and second-level analyses were also used to create a (simplified) mapping between factors and voxels. For the first-level analyses, a voxel was uniquely assigned to one of the five first-level factors for which it had the highest (absolute value) loading, provided that this loading was above a threshold value of 0.4 (a typical value for exploring factor structure). Similarly, for the second level analysis, a first-level factor was uniquely assigned to one of the 10 second-level factors for which it had the highest (absolute value) loading, provided that this loading has was above the 0.4 threshold. Considered together, the above mappings allowed us to assign a set of voxels (from different lobes and participants) to each of the second-level factors.

This assignment served the two purposes. First, it provided a basis for assessing the commonality of each factor across participants. A factor was defined as being common to N participants if it was mapped to voxels that originated in N participants. Second, the set of voxels assigned to a factor specified the brain locations associated with the factor.

The above two-level factor analysis was initially performed using data from only four of the participants, selected to optimize the discovery of semantic factors capturing neural activity across more of the cortex than just in visual areas. The four selected participants were the ones who had the greatest number of voxels with high stability in non-occipital portions of the cortex. Generally, in a task with visual input, the most stable voxels are found in occipital areas, where the stability is determined primarily by the low-level visual features of the written words. The presence of substantial numbers of stable non-occipital voxels in these four participants made it more likely that interpretable semantic factors would emerge during this initial discovery phase of analysis. The analysis was subsequently applied to all 11 participants, producing similar results, as reported below.

Although the factors emerging from a factor analysis initially have to be subjectively interpreted, we report below how the recovered factors were subjected to several validation methods. In the results section, the four emergent factors are (1) analyzed for content; (2) independently substantiated by demonstrating consistency with two other measures of word meaning; (3) used as the basis of a machine learning cross-validation protocol that demonstrates the ability to identify the word from its fMRI pattern; and (4) used as the basis of a machine learning cross-validation protocol that demonstrates the ability to predict the fMRI pattern of a new word.

### Machine Learning Methods

#### Overview

The machine learning techniques used here can be separated into three stages: algorithmic selection of a small set of voxels believed to be useful for classification; training of a classifier on a subset of the data; and finally testing of the classifier on an independent subset of the data. The training and testing use cross-validation procedures that iterate through many cycles of all possible partitionings of the data into training and testing datasets. The training set and test set are always rigorously kept separate from each other. The two main machine learning modeling approaches used are a Gaussian Naïve Bayes (GNB) classifier and linear regression. Throughout the paper, we use the term *word identification* to refer to the ability of a machine learning algorithm to determine (with some accuracy) which of many words a person is thinking about.

#### Feature selection

First, there is an algorithmic feature selection, selecting 80 of the 15,000–20,000 brain voxels (each 3.125×3.125×6 mm) believed to be particularly useful for detecting the patterns of interest. (Several previous studies indicated that 80 voxels regularly produced considerably higher identification accuracies than using all of the voxels in the brain, and modest increases of the number of voxels above 80 tended not to systematically increase accuracy.) In the base machine learning model described later, the voxels selected were the 80 most stable voxels in the cortex. Here a voxel's stability was computed as the average pairwise correlation between its 60-word activation profiles across the four presentations in a training set for the within-participant identification. The activation values for the 80 voxels were normalized (mean = 0, SD = 1) across the 60 words, separately for the training and test set, to increase comparability across the six presentations. (For the cross-participant analyses, the 80 voxels were those that were most stable across the 60 words for the participants in the training set, excluding any data from the participant involved in the test of the classifier.)

#### Classifier training

In a second stage, a subset of the data (four out of the six presentations in the within-participant classification) was used to train a classifier to associate fMRI data patterns with a set of labels (the words). A classifier is a mapping function *f* of the form: *f: voxel activation levels*→Y*_i_*, *i* = 1,…,m, where Y*_i_* were the 60 words (*leg*, *chair*, *car*, *dog*,…), and where the *voxel activation levels* were the 80 mean activation levels of the selected voxels. The classifier used here was a Gaussian Naïve Bayes (GNB)-pooled variance classifier. (Several other classifiers were also examined, such as variants of GNB-pooled, a support vector machine, and a k-nearest neighbor classifier, all of which sometimes produced comparable results. We make no claim of superiority for GNB-pooled.) GNB is a generative classifier that models the joint distribution of class Y and attributes and assumes the attributes *X_1_,…,X_n_* are conditionally independent given Y. The classification rule is:
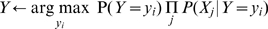
where *P(X|*Y* = y_i_)* is modeled as a Gaussian distribution whose mean and variance are estimated from the training data. In GNB-pooled variance, the variance of attribute *X_j_* is assumed to be the same for all classes. This single variance is estimated by the sample variance of the pooled data for *X_j_* taken from all classes (with the class mean subtracted from each value).

#### Classifier testing

The classifier was tested on the mean of the two left-out presentations of each word. This procedure was reiterated for all 15 possible combinations (folds) of leaving out two presentations. (The between-participant classification always left out the data of the to-be-classified participant and trained the classifier on the remaining participants' data.)

The *rank accuracy* (hereafter, simply *accuracy*) of the classification performance was computed as the normalized rank of the correct label in the classifier's posterior-probability-ordered list of classes. For example, if the classification were operating at chance level, one would expect a mean normalized rank accuracy of 0.50, indicating that the correct word appeared on average between the 30^th^ and 31^st^ position in the classifier's output of a ranked list of 60 items. A rank accuracy was obtained for each fold, and these rank accuracies were averaged across folds, producing a single value characterizing the prediction accuracy for each word. The mean accuracy across items (words) was then computed.

## Results

### Overview of Results

1. In the first section of the results, we report the outcome of a data-driven approach, a factor analysis of the brain activation, discovering three semantic factors and one visual factor underlying the representation of the 60 words that are common across participants. This section also describes the cortical locations associated with each factor.
2. We then develop converging information about the word representations by obtaining two additional characterizations that are based on (a) text corpus statistics related to the words, and (b) independent participant ratings of the words. These additional approaches indicate strong correspondences with the factor analysis characterizations of the words.
3. We then apply a machine learning (or pattern classification) approach to determine whether the semantic characterization obtained by the bottom-up approach can be used to successfully identify a word by its fMRI activation signature.
4. We show that the neural representation of a concrete noun is common across people, allowing cross-participant identification of the words.
5. We express the theory of concrete noun representation explicitly and use a regression model to test the generativity of the theory by predicting the activation of words that the model has not previously encountered and matching the predictions to the observed activation.

### 1. Using Factor Analysis to Determine the Semantic Dimensions Underlying the Activation and the Factors' Locations

#### Common factors across participants

The factor analyses start with the four participants with the greatest number of stable anterior voxels and are then generalized to the entire group because the anterior voxels encode semantic information that is part of a concrete noun's meaning. The 80 most stable voxel locations of the four participants with plentiful anterior voxels were very similar to each other and included inferior left frontal cortex, inferior parietal, and posterior temporal regions, whereas the remaining seven participants had few voxels in these anterior locations among the 80 most stable ones. We show below that the remaining seven participants also had informative anterior voxels, but there were enough stable posterior voxels among these seven participants to lower the stability rank of the anterior voxels. It was the four participants with plentiful anterior voxels (labeled P_1_, P_2_, P_3_, and P_5_ in a later Figure) who also tended to have the highest word identification accuracies using machine learning techniques.

Of the factors emerging in the second-level factor analysis, only four of them were common to all four of the participants with plentiful anterior voxels in the first-level factor analyses. (These four factors explained 29% of the variation in the input data (the 100 factors from the first-level factor analyses), whereas all 10 factors explained 56% of the variation.) The four common factors were initially interpreted by observing which words had the highest factor scores for a given factor and which had the lowest. For example, the factor we labeled as *eating-related* assigns the highest rank orders to vegetables and eating utensils. Another example is the factor labeled *word length*, which assigned the highest factor scores to the longest words and the lowest scores to the shortest words (*cat*, *cow*, *car*, *leg*, *key*), making it straightforward to interpret this factor.

There were three interesting semantic factors: *manipulation*, *eating*, and *shelter-entry*. The *manipulation* factor accords its highest scores to objects that are held and manipulated with one's hands. The 10 words with the highest factor scores for this factor included all five of the tools, as well as *key*, *knife*, *spoon*, *bicycle*, and *arm*. The 10 words with the highest factor scores for each factor are shown in Table 2. The *eating* factor appears to favor objects that are edible (all five vegetables are in the top 10) or are implements for eating or drinking (*glass* and *cup*). Note that each word has a score for each of the factors, so a word's neural representation is a composition of these four factors, such that *glass* and *cup* rank high not only in terms of *eating*, but they also have a substantial *manipulation* component (although not in the top 10). The *shelter* factor appears to favor the objects that provide shelter or entry to a sheltering enclosure. The 10 words with the highest factor scores contained three of the dwellings, four of the vehicles that include an enclosure (such as *train*), as well as *door*, *key*, and *closet*. These interpretations of the factors are consistent with converging evidence presented below. (The percentage of variation accounted for by each of the four second-level factors in the data of the four participants with plentiful anterior voxels was *eating*: 7.26; *shelter*: 8.51; *manipulation*: 7.31; *word length*: 5.67.)

**Table 2:** Ten words with highest factor scores (in descending order) for each of the 4 factors.

*Shelter*	*Manipulation*	*Eating*	*Word length*
apartment	pliers	carrot	butterfly
church	saw	lettuce	screwdriver
train	screwdriver	tomato	telephone
house	hammer	celery	refrigerator
airplane	key	cow	bicycle
key	knife	saw	apartment
truck	bicycle	corn	dresser
door	chisel	bee	lettuce
car	spoon	glass	chimney
closet	arm	cup	airplane

#### Visual features of the printed word: the word-length factor

The *word length* factor presents an opportunity to separate a low-level, perceptual feature of the printed word from the high-level, semantic object features (encoded by the *manipulation*, *eating*, and *shelter* factors). The *word length* factor appears to represent the width or number of letters of the printed word. The *word length* factor scores of each word are highly correlated with word length (r = 0.90). The locations associated with this factor (reported below) also appear to be consistent with this interpretation. There was also a check made to determine whether word frequency might also be influencing the activation at the *word length* factor locations. However, a stepwise regression on the mean activation of the voxels in each factor location determined that after having entered word length as the independent variable in the first step, entering word frequency in the second step never produced a reliable increase in R^2^ in any of the analyses of the four factor locations in any of the 11 participants. In sum, this low-level *word length* factor demonstrates that the factor analysis method can recover a factor that matches a clearly measureable property of the stimuli, and thus serves as a validity check. Moreover, the factor captures an essential part of the representation of a written word as it progresses into the semantic system.

#### Alternative analyses yielding similar results

The impact of having used the two-level factor analysis can be assessed by comparing it to a single-level analysis that finds factors in a single step (eliminating the first-level within-lobe, within-participant analyses). The single-level analysis also recovers the four factors reported above. The *shelter*, *manipulation*, and *word length* factors strongly resemble the corresponding factors in the two-level analysis (the correlations between the two sets of 60 factor scores derived from the two approaches for these three factors were 0.89, 0.92, and 0.96 respectively). However, there was a modest difference in the *eating* factor scores from the two approaches (a correlation of 0.71 between the two sets of factor scores) and, moreover, the eating factor ranked fifth among the resulting factors in the single-level analysis (having been displaced by a much less interpretable factor). The two different factor analysis approaches thus produce the same four factors. We have focused on the results of the two-level analysis because there we enforced certain assumptions (distribution across lobes and generality across participants) and because the resulting factor structure was more easily interpretable.

To confirm that the factors obtained from the four participants with plentiful anterior voxels apply well to the activation of all 11 participants, an additional two-level factor analysis was performed on all 11 participants using the method described above. The first four factors (explaining 20% of the variation in the first-level factors data) were extremely similar to the corresponding factors from the original four-participant analysis, and also were shared by a substantial proportion of the participants. The correlation between four- and 11-participant-based factor scores for the 60 words for *shelter* was .91, and the factor was present in nine of the 11 participants; for *manipulation* the correlation was .88 and the factor was present in five of 11 participants; for *eating* the correlation was .85 and the factor was present in nine of 11 participants; for *word length* the correlation was .93 and the factor was present in all 11 participants. There were other factors emerging from the four- and 11-participant factor analyses that were present in fewer of the participants than these four factors, such as a factor that could be labeled *containment*, which assigned high scores to objects capable of being filled, such as *cup* and *closet*. Thus the alternative analyses described above (the one-level factor analysis and the two-level analysis on data from all 11 participants) show that the outcomes are not closely dependent on the main methods that were used.

### Finding the Multiple Brain Locations Corresponding to Each Factor

Because the semantic factors emerge from the activation patterns of individual voxels, it is possible to trace the factors back to their root voxels and determine where the voxels associated with a given factor are located. Using the factor loading matrices from the second- and first-level factor analyses, the locations of voxels that are associated with each of the four common factors were computed from the analysis of all four participants with plentiful anterior voxels. Recall that voxels were uniquely assigned to one of the four factors by selecting their highest (absolute value) loading above a .4 threshold. For each factor, the associated voxels tended to cluster in three to five different locations in the brain. Voxel clusters were obtained by finding at least five neighboring voxels that belonged to a given factor. Then a sphere was defined at the centroid of the cluster having a radius equal to the mean radial dispersion of these voxels from the centroid.

To foreshadow, all four factors were associated with multiple locations, distributed across multiple lobes. Moreover, many of the locations associated with a given factor have been previously characterized as nodes in networks of cortical areas related to the factor in fMRI studies without verbal stimuli, as described below. Two of the factors (*manipulation* and *eating*) were very strongly left-lateralized, possibly due to handedness considerations. The *shelter* and *word length* factors included voxel clusters in both hemispheres.

**Figure 1:**
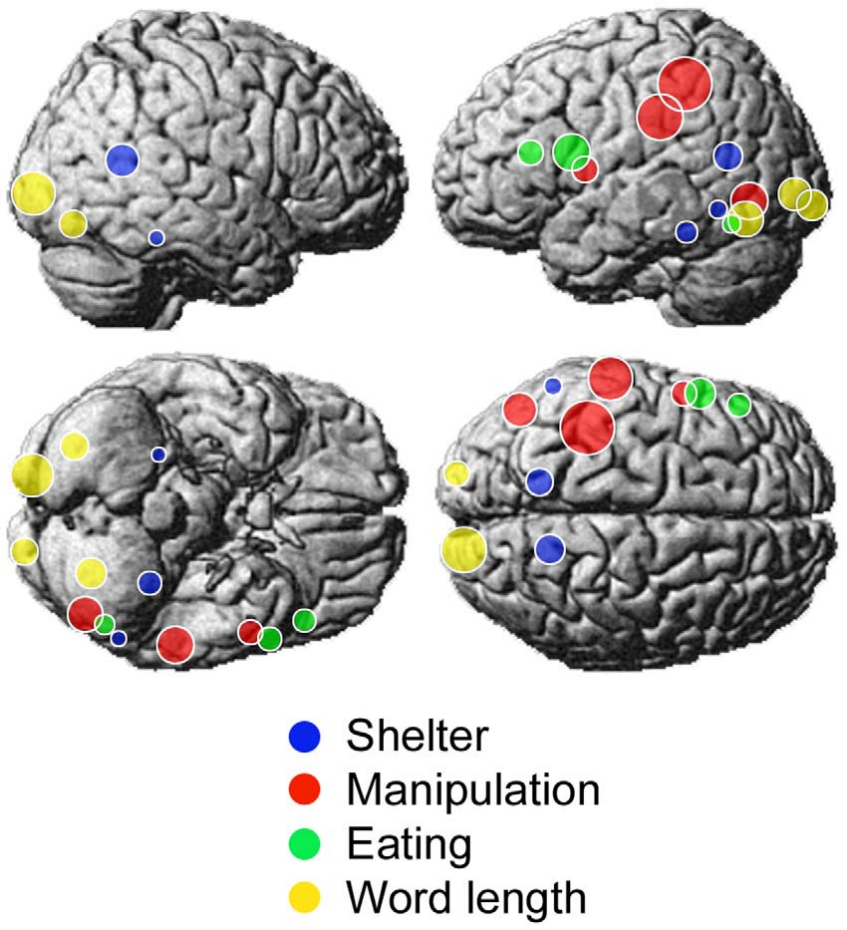
Locations of the voxel clusters (spheres) associated with the four factors. The spheres (shown as surface projections) are centered at the cluster centroid, with a radius equal to the mean radial dispersion of the cluster voxels.

The centroids and radii associated with each factor are shown in Figure 1 and Table 3. Figure 1 shows the multiple cluster locations for all of the factors in the brain as colored spheres. (Additionally, Figure S1 shows the locations of the actual voxels assigned by the above procedure to the four factors.) In the descriptions below of the correspondences between these locations and those reported in other studies, we cite the Euclidean distance from the centroid of a given cluster of a factor in the analysis above, to the peak voxel or the centroid of activation provided in the cited article. Note that the mean radius of our spheres is about 7 mm and the voxel size is 3.125×3.125×6 mm, so any centroid-to-centroid distance less than our radius constitutes an overlap of location.

**Table 3:** Locations (MNI centroid coordinates) and sizes of the voxel clusters associated with the four factors.

Factor	Cluster location	x	y	z	No. of voxels	Radius (mm)
***shelter***	L Fusiform Gyrus/Parahippocampal Gyrus (PPA)	−32	−42	−18	26	6
	R Fusiform Gyrus/Parahippocampal Gyrus (PPA)	26	−38	−20	6	4
	L Precuneus	−12	−60	16	40	8
	R Precuneus	16	−54	14	36	8
	L Inf Temporal Gyrus	−56	−56	−8	12	4
***manipulation***	L Supramarginal Gyrus	−60	−30	34	51	10
	L Postcentral/Supramarginal Gyri	−38	−40	48	21	12
	L Precentral Gyrus	−54	4	10	18	6
	L Inf Temporal Gyrus	−46	−70	−4	34	8
***eating***	L Inf Frontal Gyrus	−54	10	18	26	8
	L Mid/Inf Frontal Gyri	−48	28	18	10	6
	L Inf Temporal Gyrus	−52	−62	−14	7	4
***word length***	L Occipital Pole	−18	−98	−6	24	6
	R Occipital Pole	16	−94	0	47	10
	L Lingual/Fusiform Gyri	−28	−68	−12	20	8
	R Lingual/Fusiform Gyri	30	−76	−14	14	6

### Relating the Factor Locations to Activation Locations in Other Tasks

Even though the locations of the multiple clusters were obtained from a factor analysis of the activation in response to the presentation of printed words, many of the factor-related locations have previously been shown to activate in perceptual or motor tasks that do not involve verbal stimuli but appear to entail the same factor. Notably, the multiplicity of the locations per factor is echoed in these previous studies.

#### Manipulation factor locations

fMRI studies of object manipulation yield activation sites very similar to the multiple locations of the *manipulation* factor, according to a meta-analysis of such studies [17]. For example, the *manipulation* factor's four locations correspond extremely well (within 1.4 to 8.2 mm across the four locations) to areas that activate during actual and pantomimed hand-object interactions [18]. Similarly, three of the four locations activate during imagined grasping of tools [19]. The *manipulation* factor location in L Postcentral/Supramarginal Gyri has activated as part of a network involved in surface orientation discrimination ([20], d = 1.3 mm), object manipulation, and hand-object interaction ([21], d = 7.9 mm). The L Supramarginal area activated in hand-object interaction ([21], d = 9.5 mm) and was selectively activated during a pantomime grasping task ([22], d = 5.9 mm). L Precentral Gyrus activated in a visual pointing task ([23], d = 8.0 mm), presumably as part of the network related to visually-guided arm movement. The previous studies collectively indicate what the specializations of the separate *manipulation* factor locations might be, such the planning of motor movements, motor imagery of interaction with objects, abstract representation of motion, and lexical knowledge related to tools. Thus the *manipulation* factor appears to be decomposable in studies that focus on the components of a factor.

#### Shelter factor locations

Bilateral fusiform/parahippocampus and precuneus locations overlap well with networks of areas that activated in previous visual perception studies. The fusiform *shelter* clusters, obtained from a factor analysis of brain activation patterns in response to words, correspond well to the published “parahippocampal place area” (PPA) that activates when participants view pictures depicting buildings and landmarks [24]: the *shelter* centroids are within 2.8 mm and 4.2 mm of the PPA loci (Talairach coordinates of ((20, −39, −5) and (−28, −39, −5)) on the right and left respectively). Equally striking is the fact that four of the five *shelter* locations correspond to four areas activated when judging familiarity of pictures of places (the participant's own office or house) [25], emphasizing that the neural representation of *shelter* entails a network of areas.

The *eating* factor includes an L IFG cluster that is 4.5 mm away from the location associated with face-related actions like chewing or biting reported by Hauk et al. [2].

The *word length* factor includes bilateral occipital pole primary visual cortex clusters that most likely reflect the low-level visual representation of the printed word.

The outcome and advantage of this approach in comparison to a conventional univariate GLM analysis is presented in the Supporting Information section (Text S1). Table S1 shows the comparison of the locations of activation in taxonomic-category-based GLM contrasts to the factor locations; the GLM-derived clusters that match some of the factor locations are shown as surface renderings in Figure S2.

To summarize, the four factors, which can be localized to 16 clusters in the brain, appear to reflect the semantic and visual properties of the 60 concrete words. In many cases, there is an amazingly close overlap between the locations that encode a given factor for the 60 concrete nouns in our experiment, and areas that activate during non-verbal tasks, such as actually performing or observing hand manipulations of objects (grasping, pointing). This correspondence provides an important link between the neural representation of concrete nouns and the representation of the different types of interactions a person can have with such objects. Moreover, the multiple locations of a factor can usefully be construed as differently-specialized nodes of a network, each of which contains a representation of the object. Finally, it is important to recall that each noun is represented as a mixture of factors, such as an *apple* being both an object of *eating* and an object of *manipulation*. These findings constitute the beginnings of a neurosemantic theory of concrete noun representations, further elaborated and tested in sections below.

### 2. Relating the Semantic Factors to Other Characterizations of Word Meaning: Latent Semantic Analysis (LSA) and Independent Participant Ratings

#### Converging method 1: Latent Semantic Analysis (LSA)

One test of the interpretation of the semantic factors was obtained by using LSA (http://lsa.colorado.edu/), which applies singular value decomposition to corpus-based metrics to provide a high-dimensional (300 in our case) representation of inter-text similarity [26]. LSA was used to determine the distance between each of the 60 words and a string of five to nine words (always excluding any stimulus word) intended to correspond to each factor. The string we defined for the *eating* factor was *food vegetable meat utensil eat drink dish*; for *manipulation* it was *tool manipulate handle grip utensil*; for *shelter-entry* it was: *building dwelling residence shelter indoor enter entry drive travel*. The resulting LSA-computed distances between each stimulus word and the strings were highly correlated with the words' corresponding factor scores derived from the activation data: the correlations were, for *manipulation*: .70; *eating*: .57; and *shelter*: .46. This general type of correspondence between brain activation data and text corpus characteristics of a word was one of the main foci of the Mitchell et al. [1] analysis.

**Figure 2:**
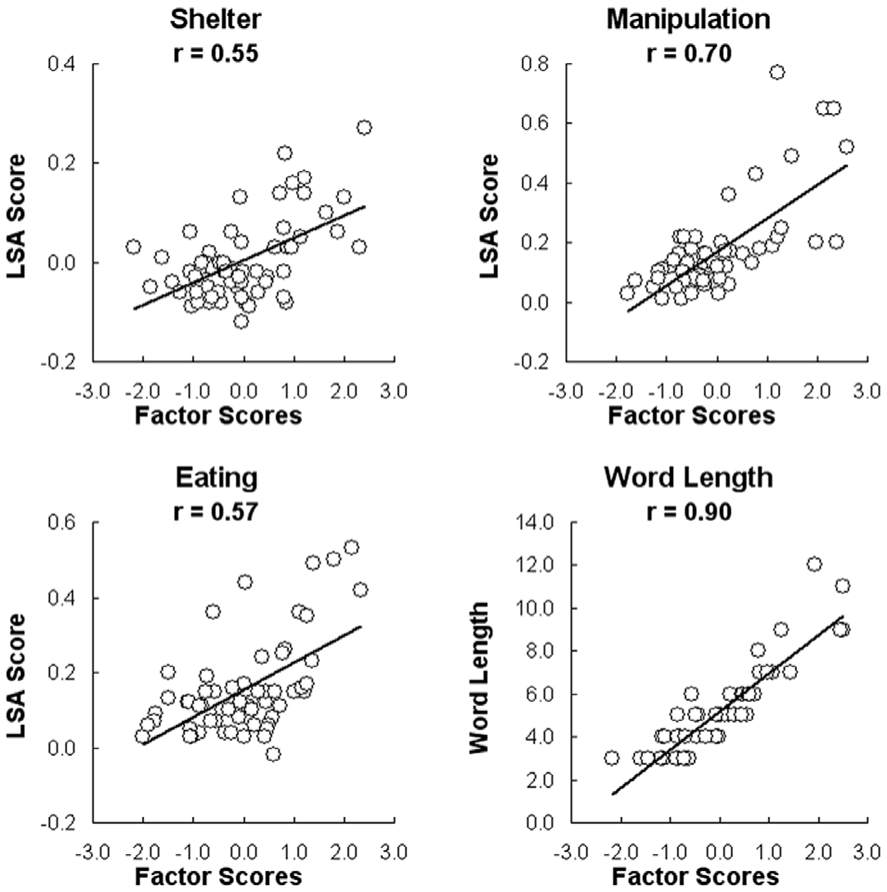
Correlation between LSA scores and activation-derived factor scores for the 60 words. For the *word length* factor, the abscissa indicates the actual word length.

Figure 2 plots these LSA distances (between the word and the factor-related-string) against the word's factor score, for each of the 60 words. The 10 rightmost points in each graph correspond to the 10 words with the highest factor scores, shown in Table 2. (Also shown is the correlation between the length of the word and the factor score, in which LSA is not involved.) These findings illustrate that an independent, corpus-based characterization of word meaning, obtained without brain imaging data, bears a substantial relation to the characterization obtained through factor analysis of the brain activation patterns.

#### Converging method 2: Independent human ratings of the words

**Figure 3:**
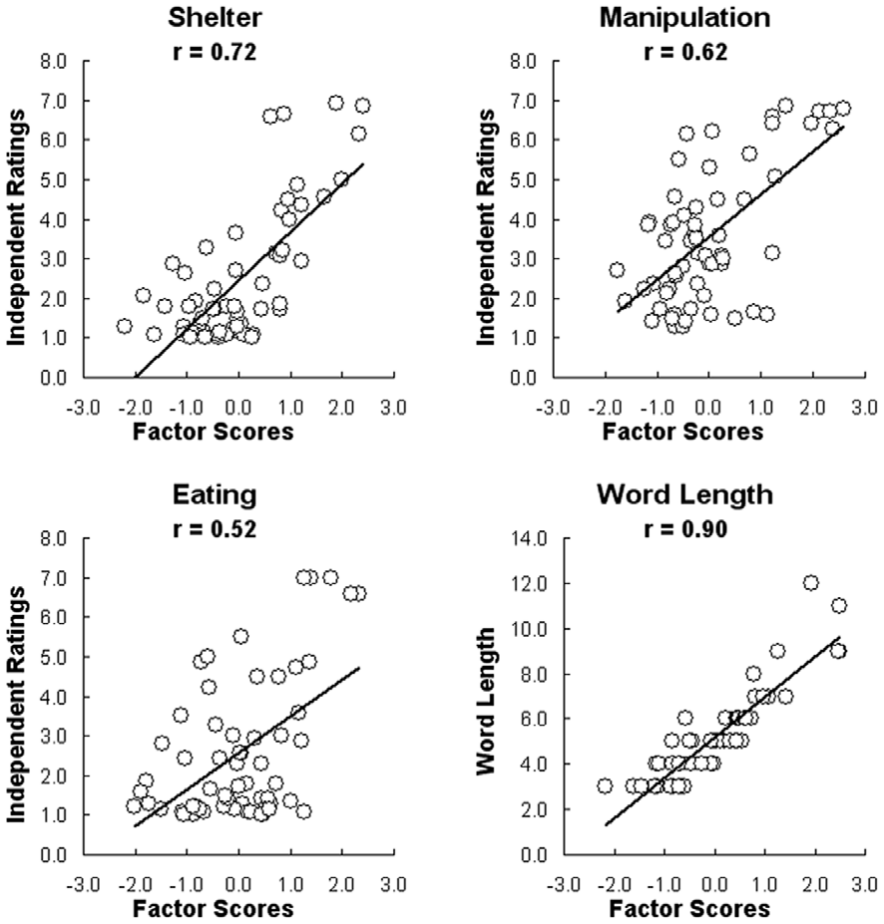
Correlation between independent ratings of the words and activation-derived factor scores for the 60 words. For the *word length* factor, the abscissa indicates the actual word length.

An independent set of ratings of each word with respect to each of the three semantic factors was obtained from a separate set of 14 participants. For example, for the *eating-related* factor, participants were asked to rate each word on a scale from 1 (*completely unrelated to eating*) to 7 (*very strongly related*). The mean ratings correlated well with the corresponding factor scores derived from the activation data: *manipulation*: .62; *eating*: .52; *shelter*: .72. For *word length*, the factor scores' correlation with the actual word length was .90. Figure 3 plots these correlations. In summary, the participant ratings of word meaning, much like the corpus-based LSA distances, provide converging information consistent with the interpretation of the factors.

### 3. Using Machine Learning (Pattern Classification) Methods to Test the Factor Approach

Although factor analysis has long been a powerful discovery tool, it often suffers from a lack of an independent method to assess the explanatory and predictive power of the analysis. To assess how well the four factors (their profiles and locations) reflect the properties of the 60 words, machine learning (ML) methods were used to construct and compare several different models of the activation. These models were first trained on a subset of the relevant data and then used to make predictions over the remaining data, enabling us to quantitatively test the accuracies of competing models. The models differed primarily in the semantic characterization that governed the selection of features (voxels).

#### Voxels selected based on semantic factors

In the ML model based on factor analysis, a feature set consisting of 80 voxels was first algorithmically selected. (Sets of voxels larger than 80 do not systematically improve the classifiers' performance.) The three properties that governed voxel selection were:

1. a semantic property (or for the *word length* factor, a visual property), namely the similarity of the voxel's mean activation profile to the profile of one of the four factors, specifically, the factor associated with one of 16 locations, described below. (The mean activation profile of the voxel is the vector of 60 mean values of the voxel's activation level for the 60 words; the factor's profile consists of the factor scores for the 60 words.) The similarity between the voxel and factor activation profiles was measured as the correlation between these two vectors.
2. a stability property, namely the stability of the voxel's activation profile over the four distinct presentations of the set of 60 words that were included in the classifier's training set. (Stability was calculated as the mean pairwise correlation between all possible pairs of the voxel's four presentation-specific activation profiles.)
3. a location property, specified by the 16 locations associated with the four factors. These locations served as the centroids of search volumes that were similar to the spheres shown in Figure 1, but larger by one voxel and shaped as cuboids, for computational simplicity

Combining the three properties above, five voxels were selected from each of the 16 search volumes, namely those five voxels with the highest product of semantic and stability scores, resulting in a feature set of 80 voxels.

To ensure independence between the training data and the test data in the ML cross-validation procedures, all of the factor-based ML analyses on a given participant used factor profiles and factor locations derived only from data from other participants. The factor profiles and cortical locations were derived from three of the four participants with plentiful anterior voxels, always excluding the participant under analysis.

### Word Identification Accuracy Based on Recovered Factors

**Figure 4:**
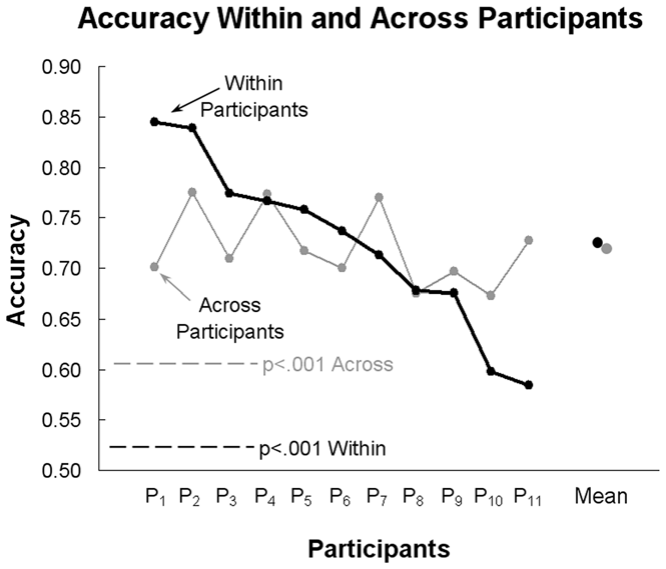
Rank accuracy of identifying the 60 individual words for each participant and the group mean. The accuracies are based on either the participant's own training set data (black) or on the data from the other 10 participants (gray), using factor-based feature selection (80 voxels) and the Gaussian Naïve Bayes classifier. The dashed lines indicate levels with p<.001 greater than chance, obtained with random permutation testing (black, within participants; gray, between participants).

The first new machine learning finding is that it is possible to identify which noun (out of 60) a person is thinking about with accuracies far above chance level by training a classifier on a subset of that person's activation data (four out of six presentations) and then making the identification over an independent dataset (the mean of the remaining two presentations). (The mean of two presentations is used simply to signal average.) This identification was based on a total of 80 voxels, five from each of the 16 locations associated with the four factors, chosen using the procedure described above. The rank accuracies of the word identification reached a maximum of .84 for two of the 11 participants (Participants P_1_ and P_2_), with a mean rank accuracy of .724 across the 11 participants. The accuracies for individual participants and the group means are shown in Figure 4 by the black curve. All of the individual participants' identification accuracies are well above chance level (the dashed horizontal black line indicates the p<.001 level of statistical difference from chance, determined by random permutation tests). These findings establish the ability to identify which word a participant is considering, based on the operating characteristics of a small set of voxels that were chosen on the basis of their match to the four factor profiles obtained from other participants' data.

Previous comparable studies of the brain activity associated with semantic stimuli have been based on the presentation of pictorial inputs (such as a sequence of photographs of physical objects from a given category, such as houses) whose visual forms were being represented (probably in an abstract form) in secondary visual processing areas, particularly ventral temporal cortex, and the activation patterns were then identified as being associated with a particular category [8]. Here, by contrast, the stimuli were printed words only, which were identified by their activation as one of 60 individual exemplars. To our knowledge, this is the first demonstration of the ability to identify the neural representation of individual words (although we have previously demonstrated the ability to do so for word-picture pairs [1]).

#### Identification within taxonomic categories

The classifier can still distinguish reasonably well even among the five words that all come from the same taxonomic category. For example, when the classifier is trained on all 60 words, the mean rank accuracy of the correct response among the five buildings (averaged over participants) is .684. The mean of such accuracies over all 12 taxonomic categories is .658, far above chance level, indicating that this method identifies more than just the category of the stimulus item. However, this accuracy is lower than when the identification is from among five randomly chosen items (which is .738), indicating that greater similarity among the alternatives decreases the identification accuracy.

#### Word identification based only on a single factor

It is interesting to ask how well words can be identified by their activation when the voxels used by the classifier are selected on the basis of only one of the factors. The accuracy was somewhat similar for the four factors used individually. The *manipulation* factor alone provided a mean accuracy of .632 (based on 20 voxels); the *shelter* factor alone led to a mean identification accuracy of .655 (using 25 voxels); the *eating* factor alone provided .593 accuracy (15 voxels); and *word length* provided .663 accuracy (20 voxels). (All of these accuracies are above the p<.001 chance level.) These results demonstrate that the factors make comparable contributions to word identification, as suggested by the similarity in the variation they each accounted for.

#### Word identification based on only the three semantic factors

If the classifier is based on only the three semantic factors (and the lower-level *word length* factor is not considered), the word mean identification accuracy was .676 (based on 60 voxels distributed among the locations of the three semantic factors), well above the p<.001 chance level and higher that any of the factors considered alone. This result indicates that *word length* contributes substantially to the .724 mean accuracy obtained when the classifier uses all four factors.

#### Machine learning using voxels selected only by stability

The semantically-based model above, which uses voxels from locations associated with the derived factors, can be compared to a baseline model that uses voxels selected only for their stability, regardless of their location within cortex. (As above, a voxel's stability is computed as the correlation of its presentation-specific activation profiles (profiles across the 60 stimulus words) across the four presentations in the training set.) The 80 whole-cortex stable voxels were located primarily in the left hemisphere (62.2% on average across participants), with a range of 43% to 80%, and were generally more posterior (visual) than the factor-based locations. This stability-only model attempts to identify which word the participant is thinking about without any consideration of word meaning, and instead characterizes only the statistical relation between the voxel activation levels and the words. The results from this model show that it is also possible to identify which noun (out of 60) a person is thinking about by selecting voxel locations simply on statistical grounds, without regard to the factor locations. The mean rank accuracy of the word identification of the stability-only model was .726 across the 11 participants. If the voxel selection procedure imposes a location constraint in addition to the stability constraint (using the 16 most stable voxels in each of five “lobes”), the mean rank accuracy is .722.

Despite the comparable accuracies of the stability-based model and the model derived from the factor analysis (in combination with stability), there are several reasons to prefer the semantic-factor-times-stability model. The first is that the selected voxels are chosen based on the mapping of their activity to a semantic factor, according them an interpretable attribute, and hence providing some face validity to the model. A second important difference is that only the semantic-factor models provide a basis for a generative theory of object representation that is extensible to new words. The model based only on stability has no capability of doing so. This facet of the theory is explored below, where semantically-based activation predictions are generated and tested for words to which the model has not been exposed. A third difference is that the voxels selected on the basis of a semantic-factor-correlation-times-stability capture important but less stable representations distributed throughout the cortex, including frontal, parietal, and temporal areas that probably encode semantic information. By contrast, the voxels selected by the baseline model, solely on the basis of stability, strongly favor posterior locations in the primary and secondary visual areas where the voxels are apparently more stable. (In the factor analysis output, these posterior voxels are associated primarily with the *word length* factor.) Thus the semantic-factor-correlation-times-stability model captures semantic representations (as well as visual representations) distributed throughout the cortex, as well as providing a basis of extensibility for the theory.

### 4. Across-Participant Word Identification

The semantic factor approach can also be used to determine whether the words have a neural signature that is common across people. The results show that it is in fact possible to identify which of the 60 words a person is viewing with accuracies far above chance level by extracting the semantically-driven neural signatures of each of the 60 words exclusively from the activation patterns of factor-related voxels of other people. The voxels were selected on the basis of their correspondence to the factors (again multiplied by stability, where stability was computed across all 10 of the participants in the training set). The model was based on the four factors and used 80 voxels. (The factor analysis that was used for selecting voxels was based on only three of the four participants with plentiful anterior voxels such that no participant's own factor analysis was used when selecting voxels for that participant's classification.) The classifier was trained on data from 10 participants and tested on the 11^th^ left-out participant (averaging first over the six presentations within a participant, and then treating the mean data from the 10 participants as though there were 10 presentations). The mean across-participant identification accuracy, averaged across the 11 participants, was .720, as shown by the gray curve in Figure 4. All of the participants' identification accuracies were well above chance level (a chance probability of p<.001 is shown by the dashed gray line). The mean accuracy for the cross-participant model was similar to the mean accuracy based on the corresponding within-participant identification, also using 80 factor-times-stability voxels (mean = .724). However, the cross-participant model had the benefit of more training data (from the 10 left out participants, averaged over their six presentations). Although the mean accuracies for the two models were similar, the cross-participant model had similar accuracies for all of the participants, whereas the within-participant model did much better on some participants than others.

The cross-participant findings provide the first evidence of a neural representation of concrete nouns based on a set of semantic factors that is common across people. This finding is an important extension of the commonality found across participants in the representation of pictures of physical objects [14].

### 5. Theory-Based Generative Prediction

The new findings can be expressed as an initial, limited theory of concrete noun representation, stating how and where a given noun is neurally represented. Specifically, each noun of the type that we studied is proposed to be represented in terms of four underlying factors at a total of 16 cortical locations, where the locations for each factor code the degree and/or nature of the relatedness of the noun to that factor. This formulation constitutes a theoretical account whose fit to the data has been described above. Below, we develop a generative or predictive account, whereby the theory is used to predict the activation of words that are not included in the data analysis.

We have recently reported a new machine learning protocol that makes it possible to measure how well a model can generate a prediction for an item (the neural representation of a particular noun) on which it has not been trained [1]. The success of any such generative approach demonstrates more than just a mathematical characterization of a phenomenon. The ability to extend prediction to new items provides an additional test of the theoretical account of the phenomenon.

In brief, two words are left out of the training set at each fold (say, *apartment* and *carrot* in one of the folds), and a regression model is trained using the data from the remaining 58 words to determine the regression weights to be associated with each of the four factors. To make the prediction, the values of the independent variables are directly derived from the ratings of the two words on the three semantic dimensions (obtained from the independent group of participants, as described above) and from the word length. Then the model can make a prediction for each of the two words, without using any information about any participant's fMRI response to those two words. The model then attempts to match the two predicted images to the two observed fMRI images for the two held-out words, based on their relative similarity to each other (using a cosine measure). There were 1,770 such attempts at matching (the number of unique word pairs that can be left out of 60 words), and the model was assessed in terms of its mean accuracy over these attempts within each participant. This approach tests whether the model developed for 58 words is extensible to two entirely new words.

To ensure that the predictive regression model had no information about the two left-out words by virtue of information from the factor analysis outcomes, a new factor analysis was run on each of the 1,770 sets of 58 words, producing a separate set of factor profiles and factor locations for each run. The underlying regression model then used the four factor profiles and the corresponding voxel locations (obtained from the data of three participants other than the one that was being analyzed).

The voxels were selected similarly to the other machine learning protocols. For each of the four factor locations (a total of 16 locations), five voxels with the highest product of the correlation with the corresponding factor profile times their stability were selected, for a total of 80 voxels. This selection procedure was performed separately for each of the 1,770 runs, leaving two words out at each iteration.

**Figure 5:**
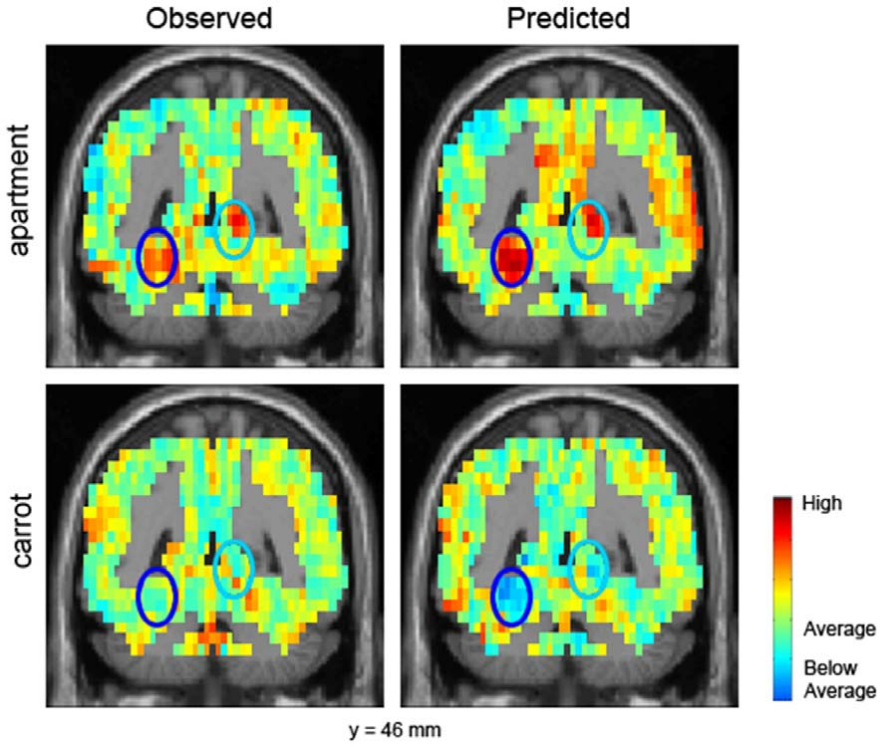
Observed and predicted images of *apartment* and *carrot* for one of the participants. A single coronal slice at MNI coordinate y = 46 mm is shown. Dark and light and blue ellipses indicate L PPA and R Precuneus *shelter* factor locations respectively. Note that both the observed and predicted images of *apartment* have high activation levels in both locations. By contrast, both the observed and predicted images of *carrot* have low activation levels in these locations.

To illustrate examples of the predictions, Figure 5 shows the presence of observed and predicted activation in the parahippocampal area and precuneus areas (indicated by dark and light blue ellipses, respectively) for *apartment*, and the absence of such observed and predicted activation for *carrot*. Analogously (but not shown in the figure), L IFG (a location for the *eating* factor) shows both observed and predicted activity for *carrot* but not for *apartment*.

The factor-based generative classification accuracy was quite high: the mean accuracy across 11 participants was .801 (far above the p<.001 greater than chance threshold of 0.537). This finding confirms that the theory concerning the neural basis of concrete noun representation is sufficiently powerful to generate predictions that successfully discriminate between new pairs of concrete nouns.

(The predictive accuracy of the regression model is not comparable to the non-generative mean rank accuracy of .724 for the classification of individual words obtained with the Gaussian Naïve Bayes classifier. The regression model attempted to answer the question “What will the activation patterns be for these two new words, given the relation between word properties and activation patterns for the other 58 words?” The Gaussian Naïve Bayes classifier attempted to answer the question of “Which of the 60 words produced this activation pattern, given information from an independent training set?”)

#### Generative prediction across participants

Just above, we demonstrated the generativity of the factor model across words. Earlier in the paper, we demonstrated the generality of the model for concrete noun representations over participants. Here we describe how both kinds of extension/generalization can be made simultaneously. The generative model can make predictions concerning two previously unseen words for a previously unseen participant. The predictions for each participant are based on data acquired from the other 10 participants for the 58 remaining words.

This factor-based cross-participant generative model matched up the two unseen words with their fMRI images with a mean accuracy of .762 across participants, which is far above the p<.001 threshold of 0.537. The theory-based model is able to extrapolate to new words while it simultaneously generalizes across participants, demonstrating the generativity of the theory.

### Comparison to a Previous Semantic Corpus-Based Model

It is interesting to consider how well a previous model (based on co-occurrence frequencies with 25 verbs of perception and action) [1] can make generative predictions based on the data from the current study. When the generative regression model was applied to the current data using the 80 most stable voxels, the accuracy for discriminating between the two left-out words was .666, compared to .801 for the factor-based generative model, a reliable difference across the 11 participants (t(10) = 5.97, p<.001.).

The relative success of the previous model speaks to the choice of verbs of interaction that were used for co-occurrence measures. The full set of verbs was *taste*, *eat*, *smell*, *touch*, *rub*, *lift*, *manipulate*, *run*, *push*, *fill*, *move*, *ride*, *say*, *fear*, *open*, *approach*, *near*, *enter*, *see*, *hear*, *listen*, *drive*, *wear*, *break*, and *clean*. These verbs are related to the semantic factors proposed here; for example, corresponding to the *eating* factor are *taste*, *smell*, and *eat*; corresponding to *manipulation* are *touch*, *rub*, *lift*, *manipulate*, *push*, *fill*, *move*, *break*, and *clean*; corresponding to *shelter* are *open*, *enter*, and *approach*.

Both the factor model and the 25-verb-co-occurrences model capture some essential characteristics of the relation between brain activation and meaning. The 25-verbs model used co-occurrences of the words with an intuitive set of 25 verbs for its characterization of the 58 modeled words and the two left-out words. The factor model that was derived bottom-up from the activation data used the resulting factors for its characterization of the 58 modeled words, and then additionally used independent participant ratings to estimate semantic values for the left out words.

A quantitative relation between the two approaches can be established by using multiple regression to determine how well each word's co-occurrences with the 25 verbs can account for the word's three semantic factor scores (separately). The co-occurrences accounted for the factor scores reasonably well (R^2^ values of .70, .65, and .59 for *shelter*, *eating*, and *manipulation*, respectively). The three verbs (among the 25) with the highest beta weights in the accounts of the *shelter*, *eating*, and *manipulation* factor scores were *near*, *fill*, and *touch*, respectively. Thus the co-occurrence measures obtained from the text corpora can to a considerable degree predict the factor scores obtained from the fMRI data.

## Discussion

The study yielded several novel findings:

1. discovery of key semantic factors underlying the neural representation of concrete nouns;
2. relating the semantic factors to brain anatomical locations;
3. accurate identification of a thought generated by a concrete noun on the basis of the underlying brain activation pattern;
4. determination of the commonality of the neural representation of concrete nouns across people; and
5. ability to predict the activation pattern for a previously unseen noun, based on a model of the content of the representation.

### Semantic Factors

The neural representation of physical objects was revealed to be underpinned by three major semantic dimensions: *shelter*, *manipulation*, and *eating*, which have several interesting properties. These dimensions have obvious face validity related to their ecological validity or survival value. It is plausible that there exist additional factors that underpin the representation of concrete nouns that were not captured by our analysis, either because of limitations of the set of stimulus words or limitations in the analysis procedures.

One limitation of the stimulus set is that it contained only count nouns (including *apple*) but no mass nouns (like *milk* or *sand*). Mass nouns cannot be grabbed or held like count nouns, requiring different types of manipulation, and hence possibly requiring a different type of representation of this factor or a different factor.

Another limitation of the stimulus set was the absence of nouns referring to human beings (there was no *sibling*, *lover*, or *attorney*). Such nouns and considerations of ecological importance suggest that there may exist one or more additional dimensions related to human interaction, with factors such as emotion and attraction.

Abstract nouns such as *kindness*, *anger*, or *innocence* were also excluded from this study. Traits and emotions seem central to the representation of such concepts, whereas *manipulation*, for example, seems less relevant. A pilot study has demonstrated that there is systematicity underlying the activation for such abstract nouns because it is possible for a classifier to identify such concepts from the corresponding brain activation with approximately similar accuracy as identifying concrete nouns. The challenge remains to relate the systematicity to some interpretable factors.

Aside from the limitations imposed by the stimulus set, there are other reasons to suspect that, even for concrete nouns, there may exist additional neural dimensions of representation. It may be that there exist other neural representational factors that are used less consistently across participants. (Recall that our analysis excluded factors observed in only a minority of the participants' data.) Two such less general factors that emerged from the analysis pertained to biological motion and to containment. The total number of semantic factors which are neurally represented may be related to the number of distinct ways that human beings can interact with an object. In this perspective, *shelter*, *manipulation*, and *eating* may simply be the most dominant factors for this particular set of stimuli.

It is also notable that the semantic factors do not directly correspond to particular visual properties of the objects to which the nouns refer. For example, neither size nor curvilinearity emerged as a factor (although it could be argued that *shelter* represents concavity and size, and the *manipulation* factor codes how one's hand might conform to an object's shape). This is not to deny that there may be a small set of visual “factors” or geometric primitives that underpin object recognition [27], which could potentially be discovered using methods like ours, but applied to visual brain area activation patterns in response to pictures of objects. It seems reasonable to assume that an object is represented in terms of both its visual properties and its semantic properties, with different tasks evoking different properties.

It is also worthwhile to note that these three dimensions are not done justice by the labels we gave them. For example, *shelter* may additionally refer to enclosure or to an allocentric frame of reference. *Manipulation* may more generally refer to physical interaction with one's body. *Eating* could possibly correspond more generally to obtaining nourishment. At the same time, some validation of these labels is provided by the success of the predictive model. That model relied on the independent participant ratings of the two left-out words with respect to these three labels in making its predictions of neural activity.

Moreover, each of the three dimensions has three to five subdimensions located at different cortical locations. Taken together, these suggest an expanded set of about 12 dimensions for the neurosemantic representation of concrete nouns (excluding the representation of the word length). Each factor appears to constitute a part of a cortical network whose constituent node specializations have been suggested by previous perceptual-motor studies, described above. Representation of all concrete nouns by voxels in about 12 locations, referred to as combinatorial coding, allows an enormous number of different individual entities to be encoded uniquely by a very modest number of voxels. In this view, there appears to be more than adequate capacity to represent all possible concrete nouns, which have been estimated to number about 1,600 concrete object types [27], as well as multiple tokens of each.

The new findings thus suggest that the meanings of concrete nouns can be semantically represented in terms of the activation of a basis set of three main factors distributed across approximately 12 locations in the cortex. Several converging methods (use of LSA and subject ratings) lend additional credence to the interpretation of the three factors. There are some indications that these three dimensions are not the only ones used in the neural representation of concrete objects. Nevertheless, the current results do reveal the beginnings of a biologically plausible basis set for concrete nouns, and they furthermore have the potential to be extended to other factors for other types of concepts.

### Brain Locations

The three semantic dimensions of representation were traced to particular sets of brain locations that have a plausible association with their interpretation. For all three semantic factors, at least some of the associated locations, derived from a factor analysis of the processing of concrete nouns, also activated in less abstract perceptual-motor tasks. The excellent matches of locations indicate that each factor corresponds to a network of cortical areas that co-activate during the factor-related processing. The previous studies also suggest that each cortical location associated with a factor is likely to be performing a distinguishable function from the other locations, although they may all be operating on a similar representation of the object.

### Identifiablity of Concepts from Activation

The new findings demonstrate the ability for the first time to accurately identify the content of a thought generated by a concrete noun in the absence of a picture, on the basis of the underlying brain activation pattern. Several alternative classifiers were comparably effective at the classification, indicating (by the way that they differ) that there is more than one set of voxels (features) that contain the relevant information. Previous studies in thought identification have presented drawings of objects [14] or object-noun pairs [1], but human thought is not limited to what we can see or hear; it extends to ideas that can be referred to in language and in other symbolic systems such as mathematics. This first demonstration of identification of symbolically-evoked thoughts opens the possibility of studying the neural representation of virtually any concept that can be communicated.

### Commonality

The results importantly revealed a commonality of the neural patterns across people, permitting concept identification across individuals. This result establishes for the first time that different brains represent concrete nouns similarly. The similarity presumably arises from a shared sensorimotor system and the shared use of the three fundamental dimensions for neurally representing physical objects. It is important to note, however, that the location and activation levels did not have to be common across people. It could have been the case that association area locations are assigned or recruited more arbitrarily. The new results indicate that not only do people have concepts in common, but also their brain coding of the concepts is similar, similar enough to decode one person's concept from other people's brain activation patterns. This is a remarkable new finding for concepts that are contemplated without visual input.

### Generative Model

The study demonstrated the ability to predict what the activation pattern would be for a previously unseen noun. Prediction goes beyond description because it entails an understanding of the underlying neurosemantic principles that relate meaning to brain activation. This demonstration of the model's generative power indicates considerable promise for extensibility to all other comparable concrete nouns. The demonstration that it performs well when trained on individuals distinct from the test subject suggests the potential for developing a general, person-independent model of word representations in the human brain (and using this as a basis to study individual differences).

### Future Questions

Although many fascinating questions are raised by these findings, we briefly mention two that seem answerable in the near future. One such question concerns the way that two or more words or concepts combine neurally to form a novel concept, such as the phrase *bird tape* or the proposition *John likes Mary*. Perhaps the methods developed here will be applicable to discovering the neural chemistry of word combinations. The other question concerns systematic individual differences in the way concepts are represented. For the participants with the highest identification accuracies, the accuracies were lower when the classifier was trained on other participants' activation, indicating that there was some systematic but idiosyncratic structure in the participant's data. It may be possible that this systematicity can eventually be understood, in terms of such possible explanations as idiosyncratic interaction with some of the objects or greater expertise in some of the object categories. Similarly, there may be systematic differences in concept representations in special populations, such that participants with autism, for example, who often have a deficit in social processing, might represent social concepts differently. Given the new ability to determine much of the content of a representation, it should be possible to determine what distinguishes the representations of individuals or special populations.

In summary, the research establishes a new way of describing brain activity, not just in terms of its anatomical location and its physical characteristics, but in terms of the informational codes that are being processed in association with a given item. Second, the work uses the underlying theory for generative prediction of brain activation, providing a set of hypothesized principles on which neural encodings of object meanings are based. These new findings not only establish new knowledge about the neural representations of meaning, but they also provide an empirical and theoretical foundation for further investigation of the content of human thought.

## Supporting Information

Text S1 Comparing factor analysis outcomes with traditional GLM contrasts for taxonomic categories.
(0.03 MB DOC)

Figure S1 Locations of the multiple voxel clusters associated with the four factors. Shelter-related voxels are shown in blue, manipulation-related voxels in red, eating-related in green, and word length in yellow.
(5.09 MB TIF)

Figure S2 Taxonomic-category-specific GLM-derived clusters that have matching factor locations. The clusters that match shelter locations are shown in blue; the cluster that matches one of the manipulation locations is shown in red, and the cluster that matches the word-length location is shown in yellow.
(1.20 MB TIF)

Table S1 Comparison of the locations of activation in taxonomic-category-based GLM contrasts to the factor locations.
(0.12 MB DOC)
