# Supplementary material for: A Neurosemantic Theory of Concrete Noun Representation Based on the Underlying Brain Codes
Source: PLoS One. 2010 Jan 13;5(1):e8622. doi: 10.1371/journal.pone.0008622 (PMC2797630; doi:10.1371/journal.pone.0008622)
Supplement: Text S1 — Comparing factor analysis outcomes with traditional GLM contrasts for taxonomic categories. (0.03 MB DOC) [file pone.0008622.s001.doc]

**Text S1**

**Comparing factor analysis outcomes with traditional GLM contrasts for taxonomic categories**

To determine whether associations between brain locations and taxonomic categories can be detected with conventional SPM GLM analyses, 12 contrasts were computed by comparing the activation associated with each category with the remaining 11 categories. Group random-effect analyses were performed using the smoothed (8 mm FWHM) normalized data using 2 x 2 x 2 mm voxels (unlike the factor analysis voxels). The maps were thresholded using a height threshold of p = 0.001 and were familywise corrected to show only the clusters that were significant at the p < .05 level (using category-specific extent thresholds that depend on the overall smoothness of the relevant SPM). Only 4 category-based contrasts showed significant clusters of activation, namely buildings, building parts, furniture, and tools (and their extent thresholds were 118, 148, 133, and 141, respectively).

The best match occurred for the “buildings” contrast, which revealed only 4 significant clusters, which closely matched 4 of the 5 *shelter* factor locations. The distances between the centroids of the GLM-derived clusters and the factor locations ranged from 5 to 7mm, indicating the consistency between the two approaches with respect to this taxonomic category, as shown in Table S1. The “building parts” contrast also yielded 4 clusters that matched the same 4 *shelter* factor locations (with inter-centroid distances ranging from 4 to 6mm), but this contrast also showed 3 additional clusters in temporal, occipital, and parietal areas that do not match any of the factor locations. The “furniture” contrast showed 4 significant clusters, one of which matches one of the *shelter* factor locations (inter-centroid distance 5mm), and 3 other clusters in occipital and cingulate areas do not match any factor locations.

The “tools” contrast yielded only one of the 4 *manipulation* factor locations (L Supramarginal), at an inter-centroid distance of 9mm. In addition, 1 cluster matches the locations of the *word-length* factor (R Lingual /Fusiform, 8 mm distal from the factor centroid), possibly because some of the tool names were particularly long. The “tools” contrast yielded 3 additional clusters that did not match any factor locations.

The GLM-derived clusters that have matching factor locations (Table S1) are shown as surface renderings in Figure S2. To facilitate the visualization aspect of the comparison of GLM-based taxonomic contrasts to the factor-based brain locations, Figure S1 shows the latter locations as surface renderings of the voxel clusters in each of the locations associated with the four factors, in contrast to the spheres shown in Figure 1.

In general, although such SPM/GLM contrasts can provide some category-specific location information for a few categories that is broadly consistent with the factor analysis outcomes, they provide no discovery procedure. To establish a GLM model, it is necessary to group items into a priori categories, such as the taxonomic categories, and then determine whether a given group of items has some neural signature. The factor analysis approach has the capability of grouping voxels that have a similar profile over the 60 words, revealing the common underlying factors and their locations. In addition, the GLM contrasts do not provide any exemplar-specific information, as the factor scores do, nor do they provide any theoretical basis for generalizing to new items.
