# Supplementary figures and images for: A Neurosemantic Theory of Concrete Noun Representation Based on the Underlying Brain Codes
Source: PLoS One. 2010 Jan 13;5(1):e8622. doi: 10.1371/journal.pone.0008622 (PMC2797630; doi:10.1371/journal.pone.0008622)

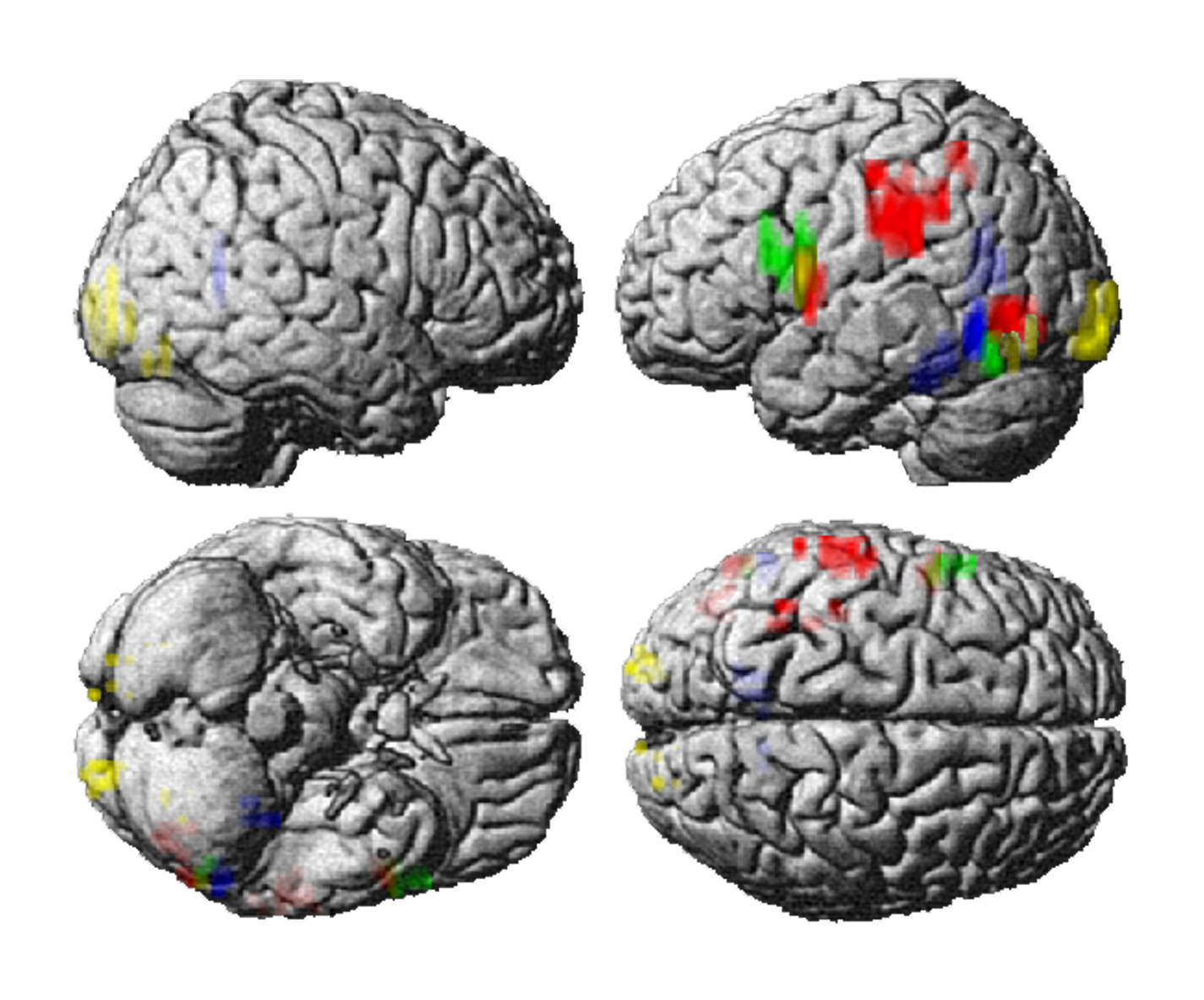

Supplement: Figure S1 — Locations of the multiple voxel clusters associated with the four factors. Shelter-related voxels are shown in blue, manipulation-related voxels in red, eating-related in green, and word length in yellow. (5.09 MB TIF) [file pone.0008622.s002.tif]

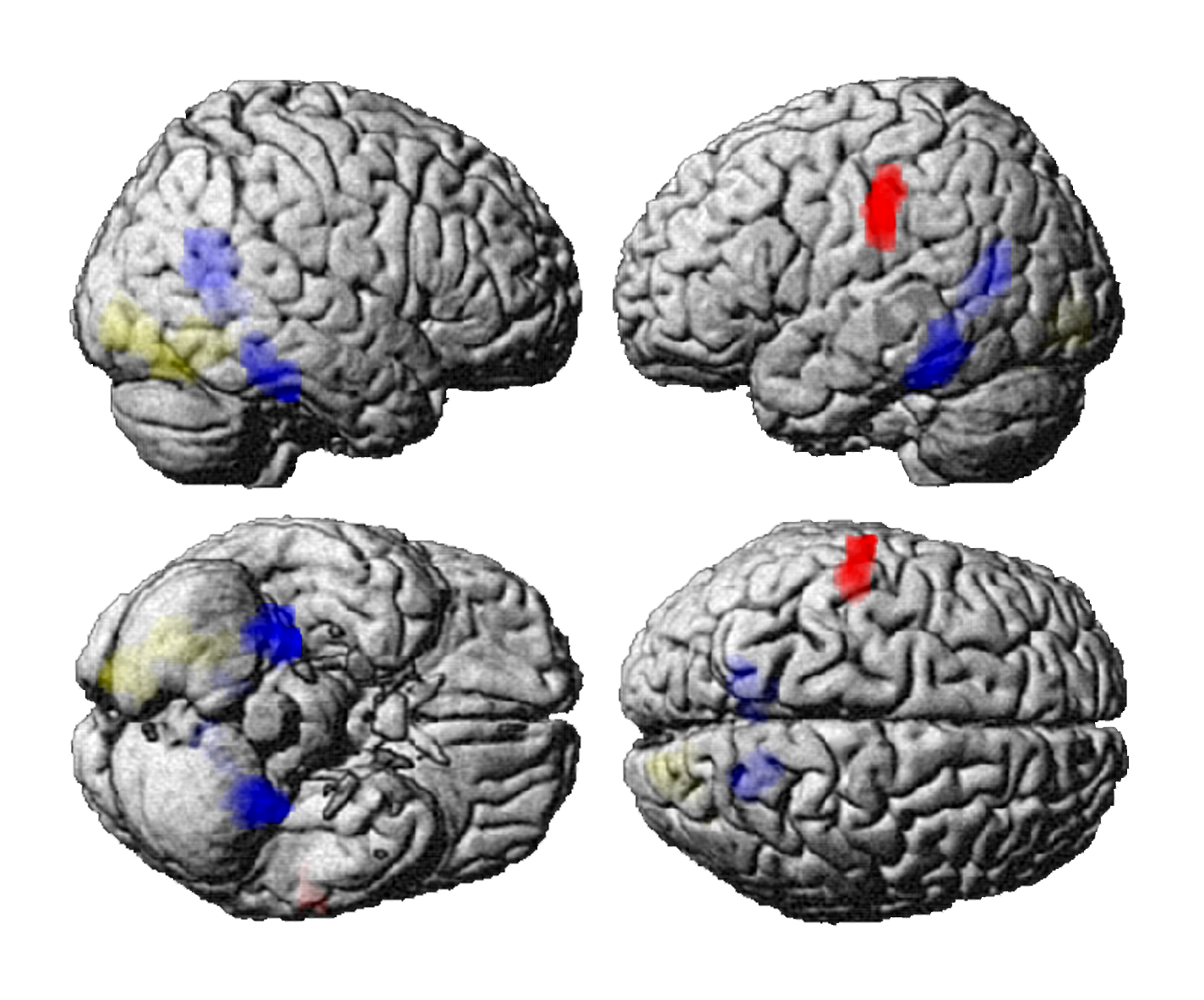

Supplement: Figure S2 — Taxonomic-category-specific GLM-derived clusters that have matching factor locations. The clusters that match shelter locations are shown in blue; the cluster that matches one of the manipulation locations is shown in red, and the cluster that matches the word-length location is shown in yellow. (1.20 MB TIF) [file pone.0008622.s003.tif]
