# Supplementary material for: A Neurosemantic Theory of Concrete Noun Representation Based on the Underlying Brain Codes
Source: PLoS One. 2010 Jan 13;5(1):e8622. doi: 10.1371/journal.pone.0008622 (PMC2797630; doi:10.1371/journal.pone.0008622)
Supplement: Table S1 — Comparison of the locations of activation in taxonomic-category-based GLM contrasts to the factor locations. (0.12 MB DOC) [file pone.0008622.s004.doc]

Table S1. Comparison of the locations of activation in taxonomic-category-based GLM contrasts to the factor locations.

**Clusters Activated by Some Taxonomic CategoriesLocation ComparisonFactors Similar to GLM Clustersbetween GLM cluster** GLM cluster locations**and Factor centroids**Factor locationsCluster centroid (MNI)Factor centroid (MNI)**Category**xyzt(peak)no. of voxelsradius (mm)**distance**

**(mm) Factor**xyzradius (mm)  **Buildings**L Precuneus-11-5896.8399 8 **7 *shelter***L Precuneus-12-6016 8L PPA-27-42-177.8338 7 **5**L PPA-32-42-18 6R Precuneus13-5487.1275 7 **7**R Precuneus16-5414 8R PPA29-34-235.8232 7 **6**R PPA26-38-20 4 **Building parts**R Precuneus19-581815.7448 8 **6 *shelter***R Precuneus16-5414 8L PPA-28-42-1610.9346 7 **5**L PPA-32-42-18 6R PPA32-39-188.1260 7 **6**R PPA26-38-20 4L Precuneus-15-62137.0231 7 **4**L Precuneus-12-6016 8R Middle Temp. Gyrus54-5206.4332 8 no matchL Occipital-36-843110.5224 7R Supramarginal Gyrus38-36466.7161 5 **Furniture**L PPA-31-43-146.1207 8 **5** } ***shelter***L PPA-32-42-18 6L Occipital-39-81297.3327 9 no matchL Middle Cingulate-5-37517.9231 7R Occipital48-74266.4170 6 **Tools**L Supramarginal Gyrus-55-233710.7409 9 **9** } ***manipulation***L Supramarginal Gyrus-60-3034 10R Lingual/Fusiform Gyri23-73-108.41039 14 **8** } ***word length***R Lingual/Fusiform Gyri30-76-14 6L Occipital-30-80011.01764 16 no matchR Occipital31-86178.0429 9L Insula-42-377.2149 7
